# Long-Term Functional Side-Effects of Stimulants and Sedatives in *Drosophila melanogaster*

**DOI:** 10.1371/journal.pone.0006578

**Published:** 2009-08-11

**Authors:** Kennedy Matsagas, David B. Lim, Marc Horwitz, Cristina L. Rizza, Laurence D. Mueller, Bryant Villeponteau, Michael R. Rose

**Affiliations:** Genescient Corporation, Corona Del Mar, California, United States of America; University of Exeter, United Kingdom

## Abstract

**Background:**

Small invertebrate animals, such as nematodes and fruit flies, are increasingly being used to test candidate drugs both for specific therapeutic purposes and for long-term health effects. Some of the protocols used in these experiments feature such experimental design features as lifelong virginity and very low densities. By contrast, the ability of both fruit flies and nematodes to resist stress is frequently correlated with their longevity and other functional measures, suggesting that low-stress assays are not necessarily the only useful protocol for testing the long-term effects of drugs.

**Methodology/Principal Findings:**

Here we report an alternative protocol for fruit fly drug-testing that maximizes reproductive opportunities and other types of interaction, with moderately high population densities. We validate this protocol using two types of experimental tests: 1. We show that this protocol detects previously well-established genetic differences between outbred fruit fly populations. 2. We show that this protocol is able to distinguish among the long-term effects of similar types of drugs within two broad categories, stimulants and tranquilizers.

**Conclusions:**

Large-scale fly drug testing can be conducted using mixed-sex high-density cage assays. We find that the commonly-used stimulants caffeine and theobromine differ dramatically in their chronic functional effects, theobromine being more benign. Likewise, we find that two generic pharmaceutical tranquilizers, lithium carbonate and valproic acid, differ dramatically in their chronic effects, lithium being more benign. However, these findings do not necessarily apply to human subjects, and we thus do not recommend the use of any one substance over any other.

## Introduction

A widespread pharmacological practice is to take a medication for a short period in order to stop a bacterial infection or relieve a symptom, such as pain. But it is now also common for patients to ingest pharmacologically-active substances for decades, whether as long-term prescribed medications, such as statins, or as over-the-counter (OTC) substances for which no prescription is needed, such as aspirin. Of particular significance are substances, or agents, that act on the central nervous system (CNS) and are taken for years or decades on a sustained basis, on the basis of a doctor's prescription, in response to medical advice, or, perhaps most often, to self-medicate.

Some of these CNS agents, such as ethanol, have long-term functional effects that are well-documented [1], [2]. Ethanol is well-known for its impairment of cognition and judgment, and also for its beneficial effects on cardiovascular disease at moderate regular doses [3]. But at sustained high doses, ethanol has health risks associated with its effects on liver function, lipids, coronary heart disease, hypertension, stroke, diabetes, dementia, and cancer progression [4], [5], [6]. In terms of aggregate effects on all-cause mortality, ethanol shows a J-shaped pattern, with overall beneficial effects on mortality at moderate consumption levels, even compared with complete abstention, yet overall deleterious effects at high levels of consumption [6]. The data that underlie this conclusion have been decades in the collection; early publications date back to the 1920s.

The problem is that the long-term effects of many other CNS agents remain unclear. For example, the relationship between coffee consumption and aggregate mortality is still unclear, despite decades of human studies [7]. A complicating factor is that the effects of coffee consumption on the mortality rates of human subjects are non-linear, sometimes gender-dependent, and apparently affected by caffeine content in a complex, sometimes ambiguous, manner.

One solution to the dilemma raised by the long-term side-effects of chronically consumed CNS agents is to study their effects in model animal species, for which regular dosing and prospective functional studies of entire cohorts throughout their lives are relatively feasible, at least compared with research on human subjects [8], [9]. However, there are problems with such studies. Of particular recent note has been the problematic status of the substance resveratrol in animal model studies. Despite promising early reports of its efficacy in several model species [10], it has proven difficult to reproduce those early results in other studies of some of these same species [11].

Life-long cohort studies of animal model species face a number of pertinent difficulties with respect to execution, analysis, and interpretation that are inherent to the use of such model species studies. The most obvious difficulty is that such animal model species are not biochemically equivalent to human subjects. However, this difficulty has been greatly alleviated with the discovery of the genomic commonalities between humans and bilaterian animal species with respect to key pathophysiological pathways [12], [13], [14]. There is a common genetic toolkit that is largely shared between humans and these model species, contrary to many preconceptions that were common for much of the 20^th^ Century, a surprising biological fact that has transformed biological research [15].

But there are a number of less obvious difficulties with life-long cohort testing that have not been resolved by the promising molecular commonality among animal species. Many of these difficulties have been reviewed in detail by Jafari and Rose [16], and we will only briefly reiterate a few key points here, as they will prove important in the interpretation of our results. It is important that model cohort studies should not employ animals that are greatly sickened either by genetic impairments, such as those produced by inbreeding and mutagenesis, or by poor experimental conditions, such as conditions in which it is difficult for animals to derive sufficient nutrition. While it might seem as if this requirement can be readily addressed simply by monitoring average longevities, this is in fact not the case.

A model organism that has been genetically sterilized or given very poor conditions can live longer because of beneficial effects of reduced reproductive activity on survival [17]. Thus a substance that is administered in an animal's food throughout their adult lives may reduce its reproductive rate, and thereby *increase* its lifespan through a reduction in the physiologically costly effect of reproduction on survival. That is, some substances may have an ostensibly beneficial effect, when only longevity or mortality-rates are monitored, an effect that can be an artifact of functional impairment of reproductive characters, characters that are often not observed in drug studies that consider only survival rates or longevity. Thus average longevity, on its own, may be a poor measure of the full spectrum of harmful effects of administered substances. In particular, to take an extreme case, a drug that sterilizes an invertebrate model organism, but thereby significantly increases its longevity, might be erroneously considered beneficial.

That this is not a hypothetical problem is illustrated well by the copious data on dietary restriction in model organisms: it is well-known that animal cohorts which receive fewer nutrients can exhibit an increase in average lifespan in conjunction with reduced fertility [18]. Chronic “medication” of an experimental cohort could have a seemingly beneficial effect if it merely reduces nutritional intake due to a perceived noxiousness of this “medication” for the model organism. Or an animal might be rendered so lethargic by a substance that its feeding rate is reduced along with other appetitive behaviors, the reduced food intake leading to reduced reproduction, with a secondary beneficial effect on longevity.

Thus it is very important for studies that use model organisms to monitor the long-term side-effects of medications on functional characters as well as aggregate measures of mortality, such as average or maximum longevity. In the present study, we present a new type of assay for the long-term effects of candidate medications, one that features a mixed-sex regime at moderate densities. In order to validate this experimental design, we performed two types of experiments.

First, we compared the mortality patterns exhibited by two well-known sets of *Drosophila melanogaster* populations that have long been shown to exhibit strikingly differentiated patterns of survival: the “B” and “O” populations long studied by Rose and his colleagues [19]. Our view was that any useful assay should replicate the much greater longevities of O flies compared to B flies.

Second, we used this assay method in studies of the chronic side-effects of four CNS agents: caffeine, theobromine, lithium carbonate, and valproic acid. These four agents were chosen as two complementary pairs. The first two are non-pharmaceutical stimulants which are commonly used by adults over their entire lifetime, caffeine being the chief stimulant found in coffee and tea, although it is also present in chocolate, while theobromine is the other key alkaloid stimulant found in chocolate. Caffeine and theobromine are similar to each other biochemically, and both are commonly perceived as “stimulants.” The second two CNS agents, lithium carbonate and valproic acid, are pharmaceutical sedatives which are prescribed for use as maintenance prophylactic medications for patients who have been diagnosed with bipolar disease, epilepsy, and a variety of related conditions. The conventional medical interpretation of their action is that they function to suppress inappropriately high level of neurological activation, although this is only a crude characterization of their complex biochemical effects [20]. Both are often taken for decades. Our view was that our assay, if valid, should be capable of revealing contrasting dose-dependent effects of these substances on lifespan and other functional characters.

## Results

### Basic Assay Protocols

#### Mortality

Stocks of *D. melanogaster* were cultured in vials with normal banana medium at about 25 degrees Celsius, as in previous assays conducted by the Rose laboratory [21]. The flies were transferred to Plexiglas cages at 14 days of age from egg and kept through adulthood at the same ambient temperature. Once in the cages, the flies were fed banana medium supplemented with yeast paste. The Petri dishes were changed daily until all the flies were dead.

#### Mating

One virgin female and two competing males, one “control” and one “treated,” were placed together in a single glass vial. Half of the treated males and half of the control males were marked with a black marker at the distal end of their right wing. The flies were given two hours in which to mate.

#### Fecundity

Laying vials were one-quarter full of charcoal agar media, with one female and one male. The number of eggs laid after one day in the vials was counted and recorded.

See the Methods and Materials for more detail.

### Using the Cage Assay to compare B and O demography

#### Demography of B and O populations

For flies derived from each of the five B and five O populations, four separate cage cohorts were used to estimate longevity and mortality rates. Each of these cohorts had very close to 750 individuals per sex, for a total of about 1,500 individuals of both sexes per cage. Thus the total number of sex-specific and cage-specific cohorts was 80. *D. melanogaster* mortality reaches a plateau at advanced ages [19], [22], at least in large cohorts, but our cohorts did not seem to be large enough to show these plateaus. Accordingly, we restricted our analysis of the individual cohorts to the Gompertz equation [23]: *A*exp(α*x*), where *A* is the age-independent rate of mortality and α is the age-dependent rate of mortality increase. The parameters *A* and α were estimated by maximum likelihood (see Methods section).

**Figure 1:**
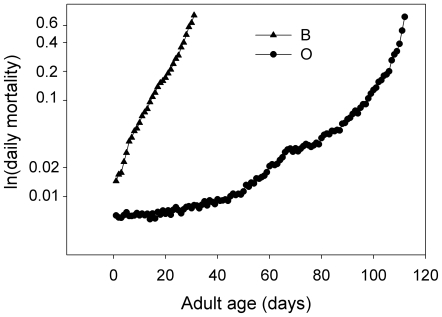
The natural log of age specific mortality in the B and O cohorts. Data of cohorts from of all populations and sexes have been combined in this figure. In total the B-mortality rates are based on 30,055 individuals and the O mortality rates are based on 29,372 individuals.

**Figure 2:**
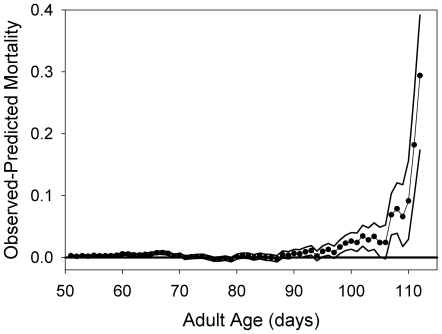
The differences between the observed mortality of the O-cohorts and the predicted Gompertz mortality (circles). The lines above and below the symbols are the binomial 95% confidence limits for the observed mortality.

The pooled natural log of mortality for the B and O populations shows no evidence of a plateau (figure 1). In fact, in Figure 1 the O mortality rates appear to accelerate at advanced ages, rather than level off. This indicates a temporal change in the cage environment that is affecting mortality, an effect that is not unexpected, because the cage environment was not cleaned during the course of the assays. Providing a new clean cage to a cohort of adult flies can require anesthetizing the entire cohort, a procedure that places them at risk of suffocating if their spiracles become clogged with medium or other material when they are immobile. For this reason, we wanted to conduct our adult mortality trials using the same cage throughout. This design choice led to visible accumulation of material on the walls of cages, much of this material being fly excreta. We thus expected to find some evidence for increased mortality resulting from the adverse effects of the deteriorating environment in the cages. To test this idea, we fit the Gompertz equation to the mortality observations and then computed the difference between the observed mortality and the predicted mortality rate (Figure 2). At later ages, the observed mortality rates become and remain larger than the Gompertz-predicted mortality. Our interpretation is that the O cage environment was becoming progressively fouled over the course of the assay, while the B flies died long before they produced such a significant fouling effect.

**Figure 3:**
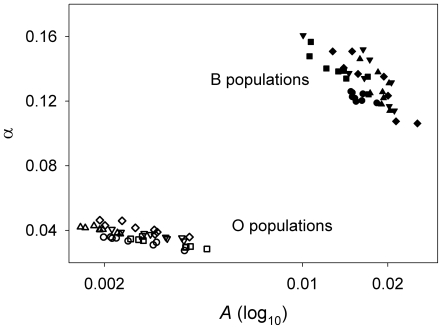
The distribution of *A* and α values for the B and O cohorts. Each symbol is data from one gender from a single population cage, e.g. males of the fourth cage of B1 flies. There are eight similar symbols representing each of the four cages and two sexes, for a total of 80 data points.

An analysis of the 80 values of *A* and α from the Gompertz equation showed no significant effect of sex on these values. A model that just included the distinct population types (i.e. B vs. O) as a fixed factor and the random factors of population and cage yielded a highly significant effect of selection regime on *A* (*p* = 4×10^−7^) and α (*p* = 5×10^−9^). There is no overlap between the B and O populations in the estimated values of these two parameters (figure 3). Population variation, such as variation among the five different B populations, contributes about 21–26% of the total variation in the two Gompertz parameters. Cage variation makes a much smaller contribution, about 1.6% of the variation in *A* and virtually none of the variation in α.

### Comparisons of CNS Drugs

All drug studies were conducted using flies derived from the B populations used in the previous study, a set of populations originally founded in 1980, and never systematically inbred [21].

We find that each of the two pairs of CNS agents studied here show strikingly different side-effects on mortality when taken throughout adult life, despite their ostensible similarities within each pair with respect to their impact on CNS functions, as either stimulants or sedatives. These findings suggest that studies of the long-term side-effects of pharmacological agents in model species may be of value particularly in revealing the potential complexity of the effects that these agents might have on chronic human health. However, we do not suggest that the specific effects of these agents on human subjects are necessarily disclosed by studies of model organisms.

#### Mortality

**Figure 4:**
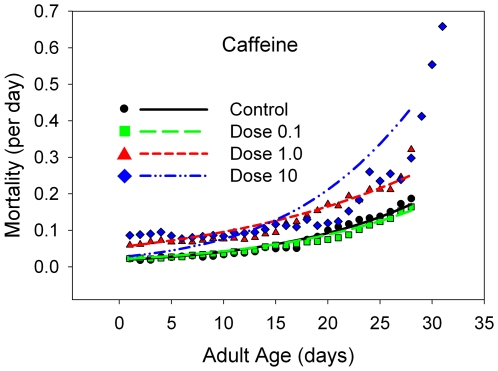
The daily mortality rates for B flies receiving a range of doses of caffeine, with a linear scale for the mortality rates. The dose levels are color-coded: black line and points, control data; yellow line and points, 10% of the estimated normal human dose; red line and points, the estimated human dose; blue line and points, 10 times the estimated human dose.

**Figure 5:**
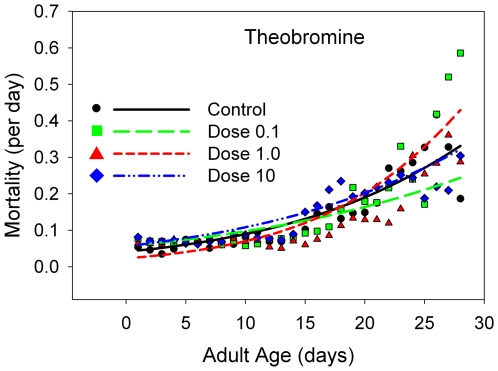
The daily mortality rates for B flies receiving a range of doses of theobromine, with a linear scale for the mortality rates. The dose levels are color-coded: black line and points, control data; yellow line and points, 10% of the estimated normal human dose; red line and points, the estimated human dose; blue line and points, 10 times the estimated human dose.

**Figure 6:**
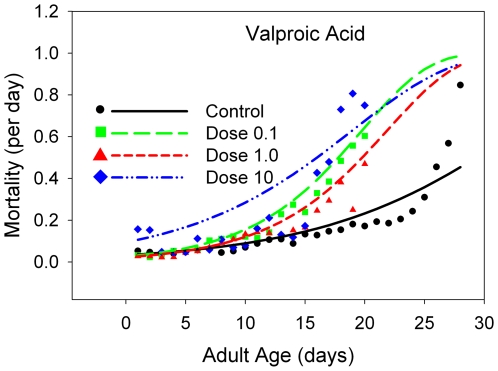
The daily mortality rates for B flies receiving a range of doses of xxxxx, with a linear scale for the mortality rates. The dose levels are color-coded: black line and points, control data; yellow line and points, 10% of the estimated normal human dose; red line and points, the estimated human dose; blue line and points, 10 times the estimated human dose.

**Figure 7:**
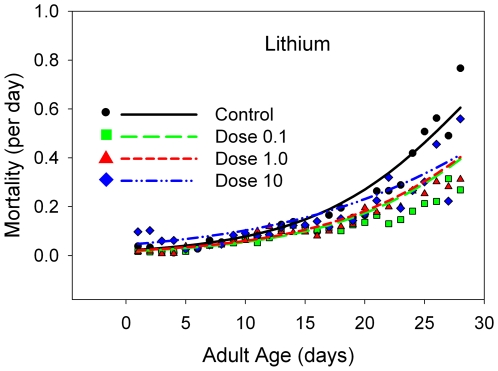
The daily mortality rates for B flies receiving a range of doses of xxxxx, with a linear scale for the mortality rates. The dose levels are color-coded: black line and points, control data; yellow line and points, 10% of the estimated normal human dose; red line and points, the estimated human dose; blue line and points, 10 times the estimated human dose.

The mean longevity is either statistically equivalent to or statistically *lower* than that at the control level for caffeine (Figure 4, table 1), theobromine (Figure 5, table 1), and valproic acid (Figure 6, table 1), but not lithium carbonate (Figure 7, table 1). At the two lowest doses of lithium, there is a slight elevation in mean longevity. An examination of age-specific mortality shows that lithium lowers both parameters of the Gompertz equation (table 2) at the two lowest doses, and the beneficial effects on the age-dependent parameter are statistically significant at the intermediate dose. The other compounds (tables 3–4, 5) typically increase one or both Gompertz parameters, leading to increased mortality rates. There are a few exceptions. Theobromine (table 3) and caffeine (table 5) significantly decrease the age-dependent Gompertz parameter but significantly increase the age-independent Gompertz parameter. These two effects work in opposite directions, and thus the longevities of flies at the lowest doses of theobromine and caffeine are about the same as that of the controls.

**Table 1:** Mean longevity (days) of adults flies receiving different doses of lithium, valproic acid, theobromine, caffeine and their controls.

Drug Dose	Mean (Days)	Standard Error
Lithium		
10	11.1	0.12
1	14.6	0.15
0.1	15.2	0.11
Control	13.4	0.31
Valproic Acid		
10	8.2	0.06
1	11.3	0.15
0.1	10.2	0.15
Control	12.4	0.15
Theobromine		
10	10.6	0.14
1	12.1	0.44
0.1	11.4	0.2
Control	12.2	0.38
Caffeine		
10	11.1	0.39
1	12.2	0.88
0.1	17.9	0.78
Control	12.2	0.38

**Table 2:** Gompertz parameter estimates for lithium.

Parameter	drug dose	Value	Std.Error	DF	*t*-value	*p*-value
Components of the age-independent Gompertz parameter, *A*						
π_1_		−4E−05	2.2E−05	482	−1.7	0.083
β_1_	control	0.0447	0.0015	482	3	0.0028
γ_11_	0.1	0.0062	0.0057	482	1.1	**0.028**
γ_12_	1	0.007	0.0051	482	1.4	0.17
γ_13_	10	0.0311	0.0055	482	5.7	<**0.0001**
Components of the age-independent Gompertz parameter, α						
π_2_		8.2E−05	7.2E−05	482	1.1	0.26
β_2_	control	0.0804	0.0048	482	1.7	0.098
γ_21_	0.1	−0.0359	0.02	482	−1.8	**0.073**
γ_22_	1	−0.0365	0.018	482	−2	**0.042**
γ_23_	10	−0.064	0.016	482	−3.9	**0.0001**

Drug doses that are significantly different from the control have bold *p*-values. The parameters are from equation (4) and indicate the following effects: density (π), controls (β), drug levels (γ).

**Table 3:** Gompertz parameter estimates for theobromine.

Parameter	drug dose	Value	Std.Error	DF	*t*-value	*p*-value
Components of the age-independent Gompertz parameter, *A*						
π_1_		−0.0001	3E−05	476	−4.5	<0.0001
β_1_	control	0.114	0.017	476	6.7	<0.0001
γ_11_	0.1	0.0325	0.0061	476	5.3	<**0.0001**
γ_12_	1	−0.026	0.0068	476	0.38	0.7
γ_13_	10	0.0241	0.006	476	4	**0.0001**
Components of the age-independent Gompertz parameter, α						
π_2_		0.0003	6E−05	476	5.2	<0.0001
β_2_	control	−0.109	0.038	476	−2.9	0.0041
γ_21_	0.1	−0.065	0.013	476	−5.1	**0.03**
γ_22_	1	−0.0205	0.013	476	−1.6	0.11
γ_23_	10	−0.0336	0.011	476	−3.1	**0.0023**

Drug doses that are significantly different from the control have bold *p*-values. The parameters are from equation (4) and indicate the following effects: density (π), controls (β), drug levels (γ).

**Table 4:** Gompertz parameter estimates for valproic acid.

Parameter	drug dose	Value	Std.Error	DF	*t*-value	*p-*value
Components of the age-independent Gompertz parameter, *A*						
π_1_		5E−06	4E−05	189	0.15	0.88
β_1_	control	0.0282	0.023	189	1.2	0.22
γ_11_	0.1	−0.007	0.0038	189	−2.5	**0.014**
π_1_		3E−05	2E−05	197	1	0.3
β_1_	control	0.0157	0.016	197	1	0.32
γ_12_	1	−0.014	0.0043	197	−3.5	**0.0007**
π_1_		−2E−05	8E−05	184	−0.31	0.75
β_1_	control	0.0446	0.052	184	0.86	0.39
γ_13_	10	0.0328	0.0187	184	1.8	0.08
Components of the age-independent Gompertz parameter, α						
π_2_		7E−06	0.0001	189	0.07	0.94
β_2_	control	0.0989	0.064	189	1.5	0.12
γ_21_	0.1	0.0772	0.012	189	6.6	**<0.0001**
π_2_		−6E−05	8E−05	197	−0.75	0.46
β_2_	control	0.143	0.0054	197	2.7	0.0085
γ_22_	1	0.0784	0.015	197	5.3	**<0.0001**
π_2_		6E−05	0.0002	184	0.36	0.72
β_2_	control	0.0722	0.11	184	0.67	0.51
γ_23_	10	0.094	0.038	184	0.25	0.81

Drug doses that are significantly different from the control have bold *p*-values. The parameters are from equation (4) and indicate the following effects: density (π), controls (β), drug levels (γ).

**Table 5:** Gompertz parameter estimates for caffeine.

Parameter	drug dose	Value	Std.Error	DF	*t*-value	*p*-value
Components of the age-independent Gompertz parameter, *A*						
π_1_		−0.000036	0.0000038	538	−9.3	<0.0001
β_1_	control	0.0601	0.0048	538	12.6	<0.0001
γ_11_	0.1	0.0131	0.0013	538	10.1	**<0.0001**
γ_12_	1	0.056	0.0035	538	16.1	**<0.0001**
γ_13_	10	0.0339	0.0037	538	9.2	**<0.0001**
Components of the age-independent Gompertz parameter, α						
π_2_		0.000021	0.000017	538	1.2	0.22
β_2_	control	0.0583	0.021	538	2.8	0.005
γ_21_	0.1	−0.0147	0.0072	538	−2	**0.041**
γ_22_	1	−0.035	0.011	538	−3.2	**0.0012**
γ_23_	10	0.0113	0.013	538	0.9	0.37

Drug doses that are significantly different from the control have bold *p*-values. The parameters are from equation (4) and indicate the following effects: density (π), controls (β), drug levels (γ).

#### Female fecundity

**Figure 8:**
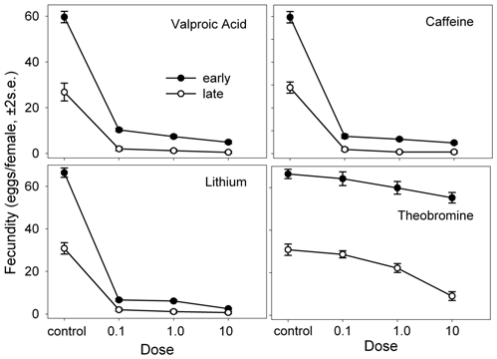
The mean fecundity of females receiving varying doses of the four drugs at two ages. The controls were maintained on normal food.

Typically these compounds all have drastic effects on female fecundity in this study (figure 8, tables 6–9). The one exception is theobromine (table 8), which does not significantly lower fecundity at the lowest dose. Any potential enthusiasm concerning the potential life extending ability of lithium is dampened by the significant negative impact of this drug on fecundity (table 9). Female fecundity shows a significant drop with age (e.g. κ is significant in every case, tables 6–9). This is of course expected due to the normal effects of aging on this life-history trait. Somewhat paradoxically, the age-by-dose interactions often show significant positive effects, suggesting that the drop in fecundity with age is not as large as expected at later ages with some substances. This effect may simply reflect the fact that at young ages these agents have already lowered fecundity so much that it drops relatively little at late ages, perhaps reflecting an “egg-hoarding” pattern.

**Table 6:** The effects of valproic acid on female fecundity.

Parameters	Drug dose	Estimate	Std. Error	*t* value	Pr(>|*t*|)
γ	control	7.69	0.071	108.2	<2×10^−16^
α_1_	0.1	−4.51	0.1	−44.9	<2×10^−16^
α_2_	1	−5.03	0.1	−50.1	<2×10^−16^
α_3_	10	−5.56	0.1	−55.3	<2×10^−16^
κ	control	−2.6	0.174	−15	<2×10^−16^
β_1_	0.1	0.579	0.246	2.35	0.019
β_2_	1	0.785	0.246	3.19	0.0015
β_3_	10	0.858	0.246	3.49	0.00054

The parameter values are from equation (9).

**Table 7 pone-0006578-t007:** The effects of caffeine on female fecundity.

Parameters	Drug dose	Estimate	Std. Error	*t* value	Pr(>|*t*|)
γ	Control	7.69	0.0725	106.1	<2×10^−16^
α_1_	0.1	−5.06	0.102	−49.3	<2×10^−16^
α_2_	1	−5.23	0.102	−51.1	<2×10^−16^
α_3_	10	−5.59	0.102	−54.6	<2×10^−16^
κ	Control	−2.33	0.177	−13.2	<2×10^−16^
β_1_	0.1	0.916	0.251	3.65	0.0003
β_2_	1	0.376	0.251	1.5	0.13
β_3_	10	0.727	0.251	2.9	0.003999

The parameter values are from equation (9).

**Table 8 pone-0006578-t008:** The effects of theobromine on female fecundity.

Parameters	Drug dose	Estimate	Std. Error	*t* value	Pr(>|*t*|)
γ	Control	8.13	0.0869	93.6	<2×10^−16^
α_1_	0.1	−0.171	0.123	−1.39	0.16
α_2_	1	−0.442	0.123	−3.6	0.00037
α_3_	10	−0.736	0.123	−5.99	4.93×10^−9^
κ	Control	−2.6	0.213	−12.2	<2×10^−16^
β_1_	0.1	−0.0187	0.301	−0.062	0.95
β_2_	1	−0.4	0.301	−1.33	0.19
β_3_	10	−1.89	0.301	−6.29	8.81×10^−10^

The parameter values are from equation (9).

**Table 9:** The effects of lithium on female fecundity.

Parameters	Drug dose	Estimate	Std. Error	*t* value	Pr(>|*t*|)
γ	Control	8.13	0.0613	132.5	<2×10^−16^
α_1_	0.1	−5.61	0.0867	−64.7	<2×10^−16^
α_2_	1	−5.69	0.0867	−65.6	<2×10^−16^
α_3_	10	−6.65	0.0867	−76.6	<2×10^−16^
κ	Control	−2.6	0.15	−17.3	<2×10^−16^
β_1_	0.1	1.36	0.212	6.42	4.11×10^−10^
β_2_	1	0.893	0.212	4.2	3.32×10^−10^
β_3_	10	1.61	0.212	7.57	2.91×10^−13^

The parameter values are from equation (9).

#### Male mating

All tests for the effects of our marking technique on mating were non-significant, so the results of our tests for marking effects are not included in our table of results. Almost all substances and all doses significantly lower the mating success of males (table 10), the one exception being theobromine, which is not significantly different from controls at the two lowest doses (table 10). There is also very often a significant age by dose interaction (table 10). This interaction indicates that these substances tend to make male performance relatively worse at old age, the opposite of the effect on female fecundity.

**Table 10:** The effects of CNS agents and age on male mating preference.

Supplement	Dose	Fraction mated to control		*p*-value for significance tests	
		early	late	dose	dosage
Caffeine	10	0.78	1	<0.00001	<0.00001
	1	0.75	0.97	<0.00001	0.00001
	0.1	0.68	0.86	<0.00001	0.006
Valproic acid	10	0.75	1	<0.00001	<0.00001
	1	0.73	1	<0.00001	<0.00001
	0.1	0.7	1	<0.00001	<0.00001
Lithium	10	0.97	1	<0.00001	0.11
	1	0.88	0.97	<0.00001	0.039
	0.1	0.82	0.98	<0.00001	0.0005
Theobromine	10	0.68	0.76	<0.00001	0.38
	1	0.59	0.54	0.01	0.71
	0.1	0.51	0.43	0.56	0.42

## Discussion

We address three issues in our discussion: (i) validity of the assay protocols that we have used; (ii) interpretation of the specific results for the substances that we have tested here; and (iii) general questions concerning the use of model systems for testing the chronic effects of substances that are used medically or recreationally for long periods by human subjects.

### Protocol Validity

Two features of the survival data were surprising for us. First, average longevities were quite low. Second, our data do not show detectable late life plateaus, a ubiquitous observation in studies of sufficiently large *Drosophila* cohorts [19], [22], including cohorts of the laboratory populations used here.

Our analysis of the low longevities starts with the premise that a valid survival assay should preserve the clear differences observed in prior studies comparing B and O cohorts. In some respects, the experiment comparing B and O populations confirmed that our protocols were valid: average longevities and estimated Gompertz parameters were all strikingly different (see Figures 1 and 3), as observed in the earlier studies. Thus our cage assays were capable of appropriately detecting differences in cohort aging patterns.

The anomaly in this extensive experimental comparison is the absence of the late-life mortality rate plateaus previously observed for these populations [19]. Figure 2 reveals why this may have occurred in the case of the long-lived O populations, which on average live more than four times longer than the B populations: our cage environments become progressively more subject to contamination. This renders age-specific mortality data essentially unreliable after the age of 80 days of adult life. However, none of our experimental tests of CNS agents involved flies surviving that long, so this contamination or “fouling” process probably did not affect our data with the B flies.

Therefore, this fouling effect does not explain the absence of a detectable late-life mortality plateau in the shorter lived B cohorts. Our explanation for this B cohort result is that the baseline mortality level, the *A* Gompertz parameter, in the present experiment is so high that not enough B flies survive long enough to reach late life. In the vial protocol used in earlier studies [19], late-life mortality plateaus were readily observed in the same type of populations as those used in the present study. But those flies lived much longer, with average longevities of more than 20 days, and average *A* parameters of 0.0034 and 0.0054 in males and females, respectively. In the present study, our controls averaged about 70 per cent of the longevities found previously [19], while the average *A* parameters were three to five times higher. In the cage environment, then, almost all the B flies don't live long enough to enter the post-aging phase. This, in several respects, makes the present cage assay protocol preferable for studies of aging specifically, in that late-life effects will have little impact on our data, rendering a Gompertz analysis relatively more reliable, at least up until the age of 80 days, when cage fouling becomes important. However, normal-lived flies will almost never survive that long. Thus we conclude that the assay methods of the present study were appropriate for substance testing with normal, as opposed to exceptionally long-lived, flies.

### Interpretation of the substance testing results

#### Lithium carbonate

Previous studies of the impact of lithium on aging in model organisms found a beneficial effect on longevity of moderate doses of lithium [24]. We were able to replicate that result here, together with progression to toxicity at higher levels of lithium. We were not surprised to find evidence for depressed male mating success and female fecundity with this substance, since it is well-known for its broadly “sedative” effects in the psychiatric literature, being a potent anti-manic agent [25]. As such, lithium provides a useful baseline that helps further validate the protocols that we have employed in this study.

#### Valproic acid

Valproic acid provides an interesting contrast with lithium carbonate. Both substances are know for their depressive effects on CNS function, and both are long-standing drugs of choice for mania, with valproic acid also in widespread use as an anti-seizure medication [26]. In keeping with such medical uses, valproic acid has broadly depressive effects on both male mating success and female fecundity in this study. Like moderate doses of lithium, moderate dosing of *Drosophila* with valproic acid reduces these two measures of functional fruit fly activity. By contrast, however, valproic acid in the formulation that we have used strikingly reduces lifespan at moderate doses, calibrated to the same dose/unit mass scale as lithium, as shown in Figure 5. Even at a dose that we estimate as equivalent to one-tenth the normal human dose, longevity is strikingly reduced, compared to both control longevity and longevity at the corresponding lithium dose. This is a marked disparity, which we discuss further below.

#### Caffeine

With caffeine, we were expecting the opposite effects on functional characters from those observed with lithium and valproic acid. That is, we expected caffeine to increase early male mating success and early female fecundity, with possible reductions in these characters later in life. We made this assumption because we expected caffeine to stimulate biological activity in *Drosophila*. This expectation was not met, and caffeine was instead generally an antagonist for the functions we tested. In addition, caffeine was clearly detrimental to survival. It was quite surprising to us to find that caffeine was such a toxic substance, at least at dose-levels that we estimate to correspond to normal human intake, in that caffeine has been tested for insecticidal activity and none has been found [27].

#### Theobromine

Like caffeine, and again contrary to our expectations, theobromine did not enhance functional activity in our flies. But, despite its biochemical and functional similarities to caffeine, theobromine was the most benign of all the substances that we tested, even at doses that we estimated to correspond to normal human levels of consumption. While there is a dose-dependent reduction in function, the effect of theobromine on both function and longevity is small relative to the effects of the three other substances that we report on here.

### Validity of using model species to test for chronic effects

Naturally, the point of greatest interest for studies of the present kind is the light that they may, or may not, shed on substances used by human subjects. Here we offer several tentative conclusions.

#### There are material discrepancies within pharmacological groupings

We find marked disparities in the effects of both supposed sedatives and supposed stimulants. Despite the parallel uses to which lithium and valproate are put, they have strikingly contrasting effects on long-term survival, at least in *Drosophila*. Likewise, caffeine and theobromine are both widely-used as stimulants, the latter usually in conjunction with caffeine in chocolate, yet in our tests caffeine proved to be strikingly toxic compared to theobromine, particularly at high doses.

These results are reminiscent of those obtained by Jafari et al [28] in their *Drosophila* studies of a group of pharmaceuticals used to treat Type II diabetes. These substances differed markedly in their chronic effects on mortality rates, some being significantly beneficial in effects on mortality, along with some heterogeneity in their effects on functional characters like those studied here. Similarly, comparisons of a large number of antioxidant substances revealed striking contrasts in their effects on aging in *C. elegans* [29].

The overall pattern that all three studies disclose is marked heterogeneity in the chronic impact of substances, at least among model species, that have been grouped together pharmacologically. Whatever intuitive notions that we might have about the chronic effects of using drugs that are commonly grouped together, such notions do not hold up when these chronic effects are tested in model organisms. How far this heterogeneity might extend is difficult to say. It is possible, for example, that slightly different pharmacological preparations may have important differences in their long-term effects on patients.

#### Functional effects may be more complex than expected

Even though caffeine and theobromine are widely used to enhance cognitive and athletic function, our results lead us to question whether these agents have all of their expected enhancing effects. For example, caffeine improves alertness and some performance tests, but it can also suppress sperm count and fertility [30], [31], [32]. The negative effects of caffeine on fertility indicate that caffeine fails to enhance Nature's most important performance – generating viable progeny. Similarly, though it is widely assumed that anti-oxidants are generally beneficial for health, because of supposed universal hazards posed by free-radical damage, the study of Lithgow and colleagues [29] suggests that such benefits are not indeed as general as widely supposed.

#### Use of model systems to test for chronic direct benefits and side effects

Finally, we come to the question of whether the use of model systems to undermine such general suppositions about efficacy is warranted. Many of the supposed benefits of agents like anti-oxidants or caloric restriction mimetics, resveratrol being a famous example, are often first identified in model organisms like *Drosophila* and *Caenorhabditis*. To the extent to which model organisms can be used to identify supposedly beneficial substances for medical or other uses, they perforce must also be of value in raising questions about the use of such agents, questions that may be directed at either the supposed benefits of such agents or their chronic side effects. The key value of analyzing the effects of compounds on life span in a model system like *Drosophila* is that long term functional effects on the whole organism can be directly evaluated. While such model-organism results should not be considered definitive in their implications for medical or recreational substance use, they can serve to focus attention on important side-effects of a chronically-used substance, ideally leading to further directed preclinical and clinical evaluation.

## Materials and Methods

### Experimental Procedures

Drug dosing: Three doses were used for each tested substance. We centered our doses on a dose that was estimated by means of a rough extrapolation from common human dosing. However, we recognized the very approximate nature of these calculations and therefore used flanking doses both ten times greater and ten times less than our estimated human dose. Our estimated daily human intake for each substance was as follows: 8 mg per kg body weight for caffeine (citrated) (CAS number 69-22-7 from Professional Compounding Centers of America), 13 mg/kg for lithium carbonate (CAS number 554-13-2 from Spectrum Chemical Mfg. Corporation), 12 mg/kg for theobromine (CAS number 83-67-0 from Spectrum Chemical Mfg. Corporation), and 10 mg/kg for valproic acid (CAS number 99-66-1 from Pharmaceutical Associates, Inc.). To extrapolate dosing, we assumed a 75 kg human and a 1 mg fly. These substances were added to yeast paste which consisted of 5 gm dry yeast, 10 mL water, and 0.5 mL acetic acid. One fourth of this yeast paste would be put on the surface of a Petri dish full of standard fly food [21]. Food plates were changed daily. We assumed that the adults would consume an amount of this yeast equal to 5% of their body weight per day. The “1.0” dose in this study corresponds to the approximate human dose and the final compound concentrations in yeast were: caffeine, 0.16 mg/gm yeast; lithium, 0.26 mg/gm; theobromine, 0.24 mg/gm; valproic acid, 0.20 mg/gm. The “10” dose was ten times these concentrations and the “0.1” dose was one-tenth of these concentrations.

#### Mortality

Stocks of *Drosophila melanogaster* were cultured in polystyrene vials one quarter full of banana agar medium at an ambient temperature of 25 degrees Celsius. The flies were transferred to Plexiglas cages at 14 days of age from egg and kept through adulthood at the same ambient temperature. The cages were custom made by Plastic Sales, Inc. in Costa Mesa, CA. The cages are made of 0.5 cm thick Plexiglas sheets sealed together with epoxy. The cages are 25 cm long, 20 cm wide and 14 cm high. On either side of the 7.5 cm by 12.5 cm opening of the cage, there is a screw with the head inside the cage and the end sticking out. A polyester sleeve with two open ends is attached to the screws through buttonholes in the seam. Another sheet of Plexiglas that that looks like a picture frame, measures 20 cm by 14 cm, and has a 7.5 cm by 12.5 cm opening for the cage and two holes for the screw ends is slid over the sleeve with the opening of the sleeve passing through the opening of the picture frame piece. This piece is held in place by two wing nuts that are screwed onto the screw ends that hold together the cage, the sleeve, and the picture frame piece. The end of the sleeve is tied in a simple knot and the flies cannot escape. Once in the cages, the flies were fed 15×100 mm Petri dishes full of banana agar medium with the surface covered in yeast paste that consists of dry yeast, acetic acid, water. In the non control cages, the yeast paste contained the assigned dosage (10, 1, 0.1 ml) of one of 4 substances: caffeine citrate, valproic acid, lithium, and theobromine. The Petri dishes were changed daily until all the flies were dead.

When fly cohorts are maintained in population cages of the design that we used, eggs laid away from the food medium fail to develop successfully, while the food medium is removed sufficiently often that development cannot be completed when the eggs are laid there. Furthermore, the pupal stage required to complete development is of sufficient duration to make the detection of successful offspring development easy to detect; no such pupal development was detected in the course of these experiments.

We set up four cages for each dosage of each of the four substances, including two sets of four control cages. Each cage contained between 1,300 and 2,500 young, mature fruit flies of the species *D. melanogaster*. Every day we supplied the cages with new Petri dishes of banana agar media, each covered in yeast paste containing the assigned substance in the assigned quantity for each cage. At this time, we counted and recorded the number of dead flies in each cage. The dead flies were removed from the cages using an aspirator. After aspiration of the dead flies from the cage, the collected flies were emptied onto a paper towel where they were counted and sexed. Male flies are identified by the presence of sex combs on the forelegs and female flies are identified by their larger, striped abdomens and the absence of the sex combs. The numbers of dead males and females were recorded in a bound notebook. It is estimated that no more than five flies per day died or escaped as a result of handling procedures. These flies were not recorded in the notebook because they did not die as a result of ingestion of their assigned substance. The above procedure continued until all of the flies in all of the cages had died.

#### Mating

We placed one virgin female, one control male, and one treated male in each of 100 glass vials for each dosage of each substance,. Half of the treated males and half of the control males were marked with a black marker at the distal end of their right wing. In the 100 vials that contained only control flies, we marked one of the males.

We gave the flies two hours in which to mate. A successful mating event was scored when a male mounted on a female for thirty seconds or more. We recorded which male was successful and whether he was marked, unmarked, treated, or control for each vial. We conducted mating assays after five days of cage life and again after three weeks.

#### Fecundity

For each dosage of each substance, and the controls, we set up 80 vials, one quarter full of charcoal agar media, containing one treated female and one treated male (or untreated in the case of the controls). The number of eggs laid after one day in the vials were counted and recorded.

We counted and recorded the number of eggs laid by the flies in each of 20 vials for each cage, 80 for each dose, after one day spent in the charcoal vials. We also conducted fecundity assays at five days of cage life and again at three weeks.

### Statistical Analysis

#### Gompertz mortality

In this formulation we will let the index *i* indicate one of the 20 cages, *j* indicates drug treatment (0 = control, 1 = 0.1, 2 = 1.0 drug, and 3 = 10 drug), and *t* indicates age. Then the predicted mortality between ages *t* and *t*+1 is *y_ijt_*. The basic nonlinear model is given by,
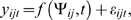
(1)where 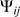 is the vector of parameters, and 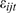 is the within population variation. The function *f* is the Gompertz model,
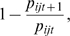
(2)where
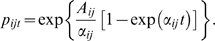
(3)


The parameter *A* is sometimes called the age-independent parameter of the Gompertz and is a reflection of background mortality that does not change with age. On the other hand α is called the age-dependent parameter and measures the rate at which mortality increases with age, e.g. senescence. We assume that the parameters of the Gompertz equation may be affected by the fixed drug treatment effect, the fixed initial cage density (*N_ij_*), and the random cage environment. These assumptions translate into a system of equations,
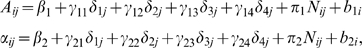
(4)where 
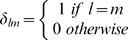
. To test for significant effects of drugs on *A* and α corresponds to a test for whether γ_1j_ or γ_2j_ is significantly different from zero [33]. The effects of different densities on *A* and α are assessed by the parameters π_1_ and π_2_ respectively.

The variance of mortality is expected to change with the mean value of mortality. The general formulation for the variance of 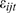 is,
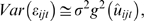
(5)where 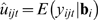. In this analysis we used *g*(.) = | *y_ijt_*|^δ^ where δ is estimated from the data. The **b** *_i_* were distributed as,
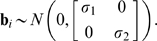
(6)


The maximum likelihood procedure would not converge when the entire valproic acid data set was run. Accordingly, we have analyzed each drug dose and control separately in table 2.

#### Demography of B and O populations

For each of the five B and five O populations, four cages were used to estimate longevity and mortality rates. Each of these cages had approximately 750 individuals per sex. We are ultimately interested in differences between the B and O populations, as well as the variation in demography that can be attributed to the replicate cages and to the replicate populations within the same selection treatment, e.g. B or O. Below we describe how we estimated the demographic parameters for each cage-sex population. From these 80 populations (4 cages×2 sexes×10 populations) we estimated variation for demographic traits and tested for significant differences between B and O populations.

We used a Gompertz model to study mortality rates [23]. This model shows exponentially increasing mortality rates at all ages. Age-specific mortality rates were modeled by the continuous-time Gompertz equation or *A*exp(α*x*), where *A* is the age-independent rate of mortality and α is the age-dependent rate of mortality increase. The parameters *A* and α were estimated by maximum likelihood.

The likelihood function was constructed from ages at death of the *N* members of a cohort following methods similar to Mueller et al. [23]. In this experiment cages were checked every day. Thus, the raw data consists of the number of dead flies recorded every day, which might be zero. We number the daily checks sequentially and let the *t_N_* be the last check during which the last fly died. Then the number of dead flies in each daily period is,
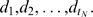



Likewise the number of flies alive at the start of each census period is *N*_1_ ( = *N*), *N*_2_, …, 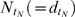. Let *q*(*i*) be the probability that an individual that lived to census period *i*, dies by census period *i*+1. Then the likelihood function is defined as,
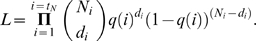
(7)The *q*(*i*) were then estimated as,
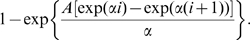
(8)


#### Female Fecundity

Female fecundity may be affected by age and drug dose. We tested this we a linear model which estimated both the effects of each drug, age and the interaction between age and drug dose. This linear model provides estimates of the magnitude of the effects of each drug and their statistical significance. Let *f_ijk_* be the number of eggs laid by the *ith* female receiving drug treatment *j* (0 = control, 1 = 0.1 drug dose, 2 = 1.0 drug dose, and 3 = 10 drug dose), and age *k* (1 = young, 2 = old). Since the numbers of eggs are very different between young and old and between some of the drug treatments, we modeled the square root of *f_ijk_* to make the variance less variable. The final model used was,
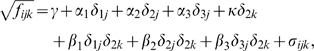
(9)where 
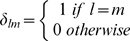
. To test for significant effects of drugs on *f_ijk_* corresponds to a test for whether α*_j_* is significantly different from zero. Similar tests on κ indicate whether there are age-specific effects on fecundity and tests on β*_j_* test for drug by age interactions.

#### Mating Preference

In this study, females were given a choice of two males to mate with. If females did not mate the experiment was discarded. Males were classified as (i) mated or not mated, (ii) marked or not marked, (iii) young or old, and (iv) drugged or controls. The counts of males in each of the possible cells from this experiment can then be analyzed by log linear hierarchical models [34]. Marking was necessary to distinguish drugged males from controls. However, since it may impact the females' preference we have controlled for this in two ways. In the mating assay, half of the controls males and half of the drugged males were marked. Therefore, we can directly test in each experiment if mating status is independent of marking status. In addition we have also competed control males against themselves with one male marked and a second male unmarked.

We consider the experiment with controls only first. We numerically identify the classification variables as mating status-1, marked status-2, and age-3. If we model the counts in each cell as simply the sum of each log of the probabilities of each factor, the appropriate statistical model is *C*_1_+*C*_2_+*C*_3_. The model term *C*_12_ indicates the sum of a two-way interaction between mating status and marked status (*C*_1∶2_) as well as the separate factors *C*_1_ and *C*_2_, i.e. *C*_1∶2_+*C*_1_+*C*_2_. Models are tested by taking the difference of the likelihood ratio, or *G* ^2^ statistic [34], of each model. This difference has a chi-squared distribution and the degrees of freedom are calculated as the difference between the degrees of freedom of the two models. Thus, a test of marking status on mating status would correspond to a test of the model with the interaction of marking and mating status (*C*_12_+*C*_3_) to the sub-model without this interaction (*C*_1_+*C*_2_+*C*_3_).

Experiments with drugged males have three questions of interest: (i) is mating status independent of drugged status, (ii) is mating status independent of marking status, and (iii) does age affect the mating status by drugged status interaction. This last hypothesis concerned whether female preference for drugged versus undrugged males changes with age. We use the same numerical labels as above, except now drug status is indicated by numerical index 4. To test hypothesis (i), we compare the model with a mating status by drug status interaction, *C*_14_+*C*_2_+*C*_3_, to one without, *C*_1_+*C*_2_+*C*_3_+*C*_4_. To test hypothesis (ii), the effect of marking on mating status, we compare the model with a mating status by making status interaction, *C*_12_+*C*_3_+*C*_4_, to a model without the interaction, *C*_1_+*C*_2_+*C*_3_+*C*_4_. To test hypothesis (iii), the possible effect of age on mate choice, we compare the model with the three way interaction between mating status, drug status and age, *C*_1∶2∶3_+*C*_14_+*C*_2_+*C*_3_, to the model without the three way interaction, *C*_14_+*C*_2_+*C*_3_.

#### Software

All statistical analyses were carried out with R (version 2.7.0 and 2.7.1, The R Foundation for Statistical Computing). The Gompertz analysis of the supplements used the non-linear, mixed effects R program (*nlme* R-package). The fecundity results were analyzed with the linear model function (*lm*) of R. The log-linear analysis of male mating was analyzed with the *loglm* R-function (*MASS* R-package). The Gompertz utilized R-code used the *optim* R-function for finding maxima of the likelihood function.
